# Beta-Arrestin1 Levels in Mononuclear Leukocytes Support Depression Scores for Women with Premenstrual Dysphoric Disorder

**DOI:** 10.3390/ijerph13010043

**Published:** 2015-12-22

**Authors:** Farzana Alam, Sanket Nayyar, William Richie, Anthony Archibong, Tultul Nayyar

**Affiliations:** Meharry Medical College, 1005 Dr. D. B. Todd Jr. Blvd, Nashville, TN 37208, USA; falam@mmc.edu (F.A.); snayyar15@email.mmc.edu (S.N.); wrichie@mmc.edu (W.R.); aarchibong@mmc.edu (A.A.)

**Keywords:** beta arrestin, mood disorders, mononuclear leukocytes, PMDD, PMS, women

## Abstract

Depression is very common in reproductive women particularly with premenstrual dysphoric disorder (PMDD), which is a severe form of premenstrual syndrome (PMS). Beta-arrestins were previously implicated in the pathophysiology, diagnosis and treatment for mood disorders. This study examined whether a measurement for beta-arrestin1 levels in peripheral blood mononuclear leukocytes (PBMC), could aid to distinguish between PMDD and PMS. Study participants (*n* = 25) were non-pregnant women between 18–42 years of age with the symptoms of PMS/PMDD, but not taking any antidepressants/therapy and at the luteal phase of menstruation. The levels of beta-arrestin1 protein in the PBMCs were determined by ELISA using human beta-arrestin1 kit. The beta-arrestin1 levels were compared with the Hamilton Depression Rating Scale scores among these women. The magnitude of the different parameters for Axis 1 mental disorders were significantly higher and beta arrestin1 protein levels in PBMCs were significantly lower in women with PMDD as compared to PMS women. The reduction in beta arrestin1 protein levels was significantly correlated with the severity of depressive symptoms. Beta-arrestin1 measurements in women may potentially serve for biochemical diagnostic purposes for PMDD and might be useful as evidence-based support for questionnaires.

## 1. Introduction

Premenstrual Syndrome (PMS) is one of the most common disorders experienced by women during their reproductive years. Up to 90% of women at their reproductive years are affected to some degree by PMS. Common symptoms of PMS include anger, irritability, and internal tension that are severe enough to interfere with daily activities. Many women have just a few mild symptoms, while others suffer severe discomfort [1]. Premenstrual dysphoric disorder (PMDD) is a severe form of PMS [2]. PMDD is usually a chronic condition and can have a serious impact on a woman’s quality of life. Unipolar depression and other Axis 1 disorders are more common in women with PMDD [3]. Treatment with selective serotonin reuptake inhibitors (SSRI) provides relief for PMDD [4]. Although drug therapies may help mask symptoms, it does little to address causative factors. The precise link between PMS, PMDD, and depression is still unclear. It is evident that women are twice as likely as men to develop major depressive disorder during their reproductive years across different countries and different settings [5].

Growing research findings suggest that G protein-receptor coupling, and its regulation, may be involved in both the pathogenesis and treatment of mood disorders [6,7,8]. Following receptor phosphorylation by G protein coupled receptor kinase, beta-arrestin binding results in desensitization of G protein-mediated signaling by preventing interaction of receptors with G proteins [9,10,11] and thereby regulates the function of many G protein coupled receptors (GPCRs), including α and β-adrenergic, muscarinic, cholinergic, serotonergic, and dopaminergic receptors [12,13]. Beta-arrestins interact with proteins of the endocytic machinery such as clathrin, to promote internalization of receptors via clathrin-coated vesicles [14,15] and are also involved in both receptor down-regulations [16] and desensitization [17,18]. A substantial body of evidence indicates that beta-arrestins that regulate G protein receptor coupling play major roles in the pathophysiology of mood disorders and in the mechanisms underlying antidepressant actions [8,19,20,21].

To study the possible involvement of beta-arrestin1 in the pathophysiology of depression associated with PMDD, we undertook measurement of beta-arrestin1 protein in mononuclear leukocytes from women participants at luteal phase of menstrual cycle to support the results obtained with questionnaires.

## 2. Methods

### 2.1. Participants

Non-pregnant women between the ages of 18–42 years, with symptoms of PMS/PMDD at luteal phase of menstruation, were evaluated with the Neuropsychiatric Interview for Axis I Diagnostic and Statistical Manual of Mental Disorders, 4th edition, criteria (DSMIV-TR) by psychiatrists [22]. The severity of depression was determined by the 17-item Hamilton Rating Scale for Depression (HAM-D); >19 = depression. Inclusion criteria were (1) 18–42 years old non-pregnant women at luteal phase with PMS; (2) good general health with no clinically significant systemic abnormalities and no major findings from a physical examination; (3) no treatment with antidepressants for last 4 weeks; and (4) willing and able to give informed consent. Exclusion criteria for all subjects were: (1) history or evidence of clinically significant physical disorders; (2) diagnosis of a major psychiatric disorder other than a major depressive disorder; (3) past diagnosis of schizophrenia, bipolar or primary anxiety disorders; (4) women suffering from dysthymia; (5) any antidepressant/psychotropic or substance use within the past 4 weeks other than caffeine, nicotine; and (6) under any medications that will present with depressive symptoms (judged from the medical records). After complete description of the study to the participants, written informed consent was obtained for a 20 mL blood donation. The Hamilton depression scale was administered before blood donation. The study was approved by the Institutional Review Board, Meharry Medical College. A total of 25 participants (22 black and 3 white) were recruited for this study.

#### HAM-D

The participant’s HAM-D score was calculated on the basis of their answers on the following areas in the questionnaires (where, 0 = absent): Depressed mood (gloomy attitude, pessimism about the future, feeling of sadness, tendency to weep): 1 = sadness, 2 = occasional weeping, 3 = frequent weeping, 4 = extreme symptoms; Difficulty in work and activities: 1 = feelings of incapacity, listlessness, indecision, 2 = loss of interest in hobbies, decreased social activities, 3 = productivity decreased, 4 = unable to work; Agitation (restlessness associated with anxiety), Insomnia early (difficulty in falling asleep), Insomnia middle (complains of being restless and disturbed during the night) and, Insomnia late (waking in early hours of the morning and unable to fall asleep again): 1 = occasional, 3 = frequent; Psychological anxiety: 1 = tension and irritability, 2 = worrying about the minor matters, 3 = apprehensive attitude, 4 = fears; Somatic anxiety (gastrointestinal, indigestion, cardiovascular, palpitation, headaches, respiratory, genito-urinary, *etc.*): 1 = mild, 2 = moderate, 3 = severe, 4 = incapacitating; Somatic symptoms (loss of appetite, heavy feeling in abdomen, constipation): 1 = mild, 2 = severe [23].

### 2.2. Isolation of Mononuclear Leukocytes

The BD vacutainer cell preparation tubes with sodium citrate (CPT) were used to collect blood samples using venipuncture technique. Isolation of mononuclear leukocytes was performed according to the manufacturer’s instructions. Briefly, The CPT tubes were inverted gently to mix the blood with anticoagulant additive and then centrifuged (Thermo Scientific 1-liter general purpose centrifuge) at 1500× *g* at room temperature (18–25 °C) in a horizontal rotor (swing-out head) for a minimum of 20 min. Using a Pasteur pipette, the mononuclear cells (the white buffy coat under the top plasma layer) were gently collected to a 15 mL tube and washed with phosphate buffered saline (PBS), centrifuged at 400× *g* for 15 min. The supernatant was discarded. Again PBS was added and centrifuged at 400× *g* for 10 min. The cell pellets were stored at −80 °C until further use.

### 2.3. ELISA

The samples were extracted using T-PER extraction reagent (Pierce BioTechnology; Rockville, IL, USA) in the presence of Halt protease inhibitor cocktail (Pierce, Rockford, IL, USA) at 10 µL/mL; sonicated for one minute, and then centrifuged at 800 x g for 5 min. The resultant supernatant was then aliquoted and stored at −80 °C for ELISA. Beta arrestin1 levels were measured in the extracts by ELISA protocol supplied by the manufacturer of the antibody (BlueGene ELISA Kits Life Sciences Advanced Technologies, Inc., FL, USA, Human Arrestin Beta 1 (ARRb1) ELISA KIT Catalogue Number: E01A0344). All assays were done in duplicates and measured on the Enoch microplate reader. Beta-arrestin1 levels in each sample were calculated by reference to a standard curve constructed for each assay. Protein content of the samples used was determined by Bradford assay. The beta-arrestin1 levels in each sample (in pg/mL) were then adjusted for the protein content of each sample, to give a final beta- arrestin1 result expressed as pg per mg of protein.

### 2.4. Statistical Analyses

All calculations and statistical analysis were carried out using Excel and GraphPad Prizm software (Graph-Pad Software, Inc., San Diego, CA, USA). Unpaired students *t*-tests were used to test for significance of association; a statistically significant result was one in which *p* < 0.05. Results are expressed as mean ± standard error.

## 3. Results

**Figure 1:**
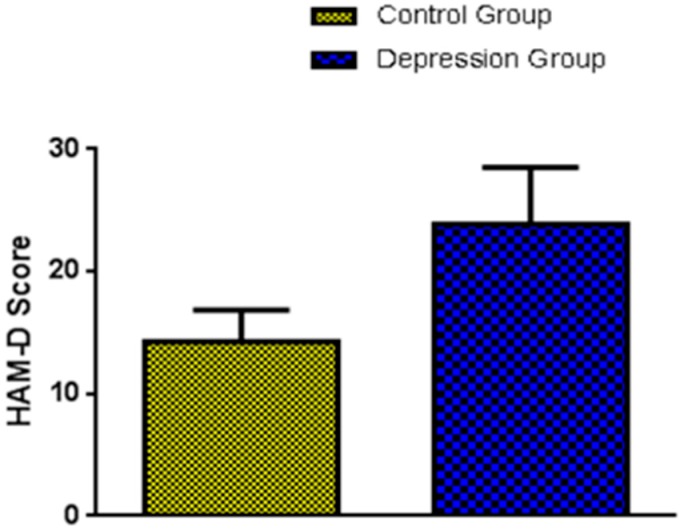
Participants were divided into two groups: depression group (HAM-D >19) and control group (HAM-D <19). Depression (*n* = 12, 11 black and 1 white) and control (*n* = 13, 12 black and 1 white) group.

To rate the severity of depression symptoms in women with PMS/PMDD, HAM-D scores assessment were done for every participant. In this study, these women met the criteria for depression at a single time point in the luteal phase of their cycle. The higher the HAM-D score, the more severe is the depression [22]. Based on the HAM-D score, the participants were divided into two groups: depression group (HAM-D >19) and control group (HAM-D <19). Out of 25 women recruited in the study, 12 women were diagnosed with depression (11 black and 1 white) and 13 women (12 black and 1 white) with non-depression which thus served as control. The mean values with the standard error of mean (SEM) for the HAM-D scores were 23.83 ± 1.35 and 14.31 ± 0.71 for depression and control group, respectively (Figure 1).

**Figure 2:**
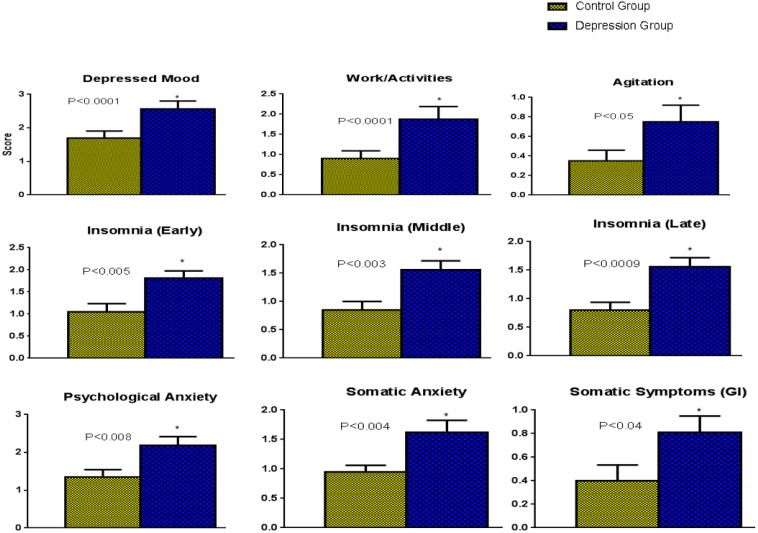
The comparison of the different items on the HAM-D scale between depression and control group of participants.

Figure 2 shows significant differences on most of the items on the HAM-D scale between depression group and control group. The depression group scored significantly high on: depressed mood (*p* < 0.0001), difficulty in work and activities (*p* < 0.0001), agitation (*p* < 0.005), and insomnia early (*p* < 0.005), insomnia late (*p* < 0.0009), psychological anxiety (*p* < 0.008), somatic anxiety (*p* < 0.005); and to lesser extent on the differences in insomnia middle (*p* < 0.003) and somatic symptoms (*p* < 0.05).

The beta-arrestin1 levels were determined blind and were compared with the independently obtained HAM-D score. All assays were done in duplicates. Standard curves and the %CV for the duplicate samples were all within acceptable range for each assay. The mean values with the SEM for the beta-arrestin1 levels in the leukocytes, expressed as pg/mg protein (Table 1), were 96.12 ± 2.45 in participants with the HAM-D scores of 23.83 ± 1.35 (depression group) and 106.7 ± 2.14 in those participants with the HAM-D scores of 14.31 ± 0.71 (control group).

**Figure 3:**
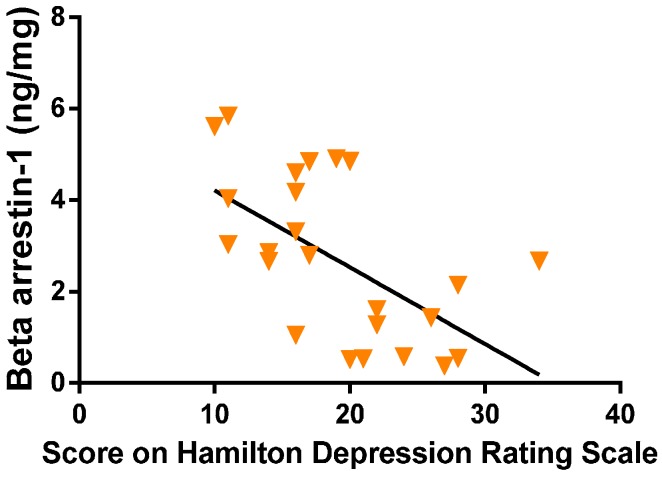
Correlation between beta-arrestin1 levels and the HAM-D score.

These data demonstrates that beta-arrestin1 levels in the leukocytes of depression group were significantly reduced (*p* < 0.005) as compared to the control group. The degree of reduction in mononuclear leukocyte beta-arrestin1 levels was found to be significantly correlated with the severity of the depressive symptoms as determined by the Hamilton depression scale score (Figure 3).

**Table 1:** The beta-arrestin1 levels in the leukocytes were compared with the independently obtained HAM-D score.

Participants HAM-D Score Beta-arrestin1 (pg/mg Protein)		
Control group	14.31 ± 0.71	106.7 ± 2.14
Depression Group	23.83 ± 1.35 *	96.12 ± 2.45 *

* *p* < 0.005.

## 4. Discussion

The hallmark feature of PMS and PMDD is the cyclic nature of symptoms that begins in the late luteal phase of the menstrual cycle and remits shortly after the onset of menstruation. The clinical symptoms of PMDD are similar to those of PMS, but are severe enough to interfere with work, social activities, and relationships. In both PMS and PMDD, underlying depression and anxiety are common [19]. The role of beta-arrestin1 protein in the pathophysiology of depression has been implicated. The beta arrestin1 protein levels in mononuclear leukocytes of untreated patients with depression were reported to be significantly lower when compared to healthy subjects [8,24]. In order to determine biomarker for PMDD, we hypothesize that beta-arrestin1 protein levels may be reduced in women with PMDD as compared to PMS, at the luteal phase of menstrual cycle. Participant and Clinical Interactions Resource (PCIR) at Meharry Clinical Research Center, identified the study participants by putting up flyers, posting advertisements in approved Meharry publications and through recommendations from existing participants in similar studies who had friends or family members who might be eligible. Non-pregnant women (menstrual cycle length 25–28 days) who were on their menstrual cycle phase on any day between days 21–28, the highly susceptible period for PMS/PMDD, were recruited on voluntary basis for this study. Two months prior to scheduling appointment, interested women were adequately informed about the research study protocol in which they were asked to enroll, including to maintain a menstrual cycle diary.

Our findings (Figure 3) from a total of 25 women, participated in this study with PMS or PMDD (severe PMS), show that PMDD women had HAM-D scores >19 (*n* = 12) and were having significantly reduced levels of beta-arrestin1 protein in the PBMC as compared to the other women (*n* = 13) with HAM-D scores <19 (PMS). Although regular PMS and PMDD both have physical and emotional symptoms, PMDD causes extreme mood shifts. PMDD occurs in three to five percent of menstruating women. Women with a personal or family history of mood disorders—including major depression or postpartum depression—are at greater risk for developing PMDD [25]. Unipolar depression and other Axis 1 disorders are more common in women with PMDD [3]. Since PMDD is distinguished from PMS by the severity of symptoms, predominance of mood symptoms, and role dysfunction, we have compared the data on the different areas on the HAM-D scale between these two groups of women as based on the HAM-D scores (Figure 2). Our findings show that: depressed mood, difficulty in work and activities, agitation, insomnia early, insomnia middle, insomnia late, psychological anxiety, somatic anxiety and somatic symptoms were significantly high in the women having HAM-D scores >19 (designated as “depression group”) and therefore, may be referred to as women with PMDD.

Most researchers believe that PMDD is brought about by the hormonal changes related to the menstrual cycle [19]. Women with PMDD are differentially sensitive to their natural hormone changes. Earlier studies have shown a connection between PMDD and low levels of serotonin [25,26,27,28,29] and as such treatment with selective serotonin reuptake inhibitors, which increases the serotonin level, provides relief for PMDD [4]. It is well evident that ovarian hormones are integral to serotonin neurotransmission [30,31,32] which involves G protein coupled receptor (GPCR) signaling [33]. GPCR signaling is well known to be involved in both the pathogenesis and treatment of mood disorders [8,13,20]. The beta-arrestin1 protein plays pivotal role in the regulation of serotonin neurotransmission via GPCR [34] and also in estrogen mediated neuroprotection [21]. Our findings show that reduction in beta-arrestin1 level was significantly correlated with the results from depression scoring (Figure 3). It has been reported previously that beta-arrestin1 protein and mRNA levels in mononuclear leukocytes of untreated patients with major depression were significantly lower than those of healthy subjects. Furthermore, reduced levels of beta-arrestin1 protein and mRNA were significantly correlated with the severity of depressive symptoms [8,34]. However, the low beta-arrestin1 protein and mRNA levels were alleviated by antidepressant treatment. Normalization of beta-arrestin1 measures preceded, and thus predicted clinical improvement [8,35]. These clinical data suggest that assessment of beta-arrestin1 levels may prove useful for diagnosing depression with high sensitivity and specificity [36].

## 5. Conclusions

Since PMDD is a severe, sometimes disabling extension of PMS, these data support the implication of beta-arrestin1 in the pathophysiology of PMDD. Beta-arrestin1 measurement in women with PMDD may potentially serve as biomarker for PMDD and might be useful as evidence-based support for questionnaires.
